# The Expression of PPAR Pathway-Related Genes Can Better Predict the Prognosis of Patients with Colon Adenocarcinoma

**DOI:** 10.1155/2022/1285083

**Published:** 2022-04-18

**Authors:** Xiao-Yu Zhou, Jian-Qi Wang, Jin-Xu Chen, Jing-Song Chen

**Affiliations:** Department of Gastrointestinal Surgery, The First Affiliated Hospital of Guangzhou Medical University, Guangzhou 510120, China

## Abstract

The postoperative survival time and quality of life of patients with colon adenocarcinoma (COAD) varies widely. In order to make accurate decisions after surgery, clinicians need to distinguish patients with different prognostic trends. However, we still lack effective methods to predict the prognosis of COAD patients. Accumulated evidences indicated that the inhibition of peroxisome proliferator-activated receptors (PPARs) and a portion of their target genes were associated with the development of COAD. Our study found that the expression of several PPAR pathway-related genes were linked to the prognosis of COAD patients. Therefore, we developed a scoring system (named PPAR-Riskscore) that can predict patients' outcomes. PPAR-Riskscore was constructed by univariate Cox regression based on the expression of 4 genes (NR1D1, ILK, TNFRSF1A, and REN) in tumor tissues. Compared to typical TNM grading systems, PPAR-Riskscore has better predictive accuracy and sensitivity. The reliability of the system was tested on six external validation datasets. Furthermore, PPAR-Riskscore was able to evaluate the immune cell infiltration and chemotherapy sensitivity of each tumor sample. We also combined PPAR-Riskscore and clinical features to create a nomogram with greater clinical utility. The nomogram can help clinicians make precise treatment decisions regarding the possible long-term survival of patients after surgery.

## 1. Introduction

Colon adenocarcinoma (COAD) is the world's second most prevalent cancer and the third greatest cause of cancer-related death [1]. Despite the fast advancement of COAD surgery and adjuvant therapy, 65 percent of patients with severe COAD will experience recurrence and metastasis, with a 5-year survival rate of less than 10% . As a result, effectively forecasting the prognosis of COAD patients and distinguishing between high-risk and low-risk patients has emerged as one of the most pressing clinical issues. Patients with low survival prospects can benefit considerably from prompt follow-up and the adoption of effective treatment regimens. For patients with a greater chance of survival, less intrusive testing and lower chemotherapy medication dosages can enhance quality of life. Although blood biomarkers like CA19-9 (carbohydrate antigen 19-9) and CEA (carcinoembryonic antigen) are often employed in COAD diagnosis and prognosis, their sensitivity and specificity are low. We still need to seek for more reliable indicators to predict the prognosis of patients with CC, which will help guide clinical care while also providing new opportunities for exploring novel therapeutic targets.

Peroxisome proliferator-activated receptors (PPARs) are a group of nuclear receptor proteins discovered in 1990. They exist in three different subtypes, PPAR*α* (NR1C1), PPAR*β*/*δ* (NR1C2), and PPAR*γ* (NR1C3), and can act as ligand-activated transcription factor (TF) [3]. Recent research has demonstrated that PPARs have an anticancer effect in addition to their metabolic efficiency [4–5], [2, 6]. Furthermore, a number of studies have discovered that abnormal PPARs and PPAR target gene expression are frequently related to the development and progression of cancer [7–9], and agonists of the PPAR pathway, such as thiazolidinediones, are typically regarded antiproliferative in tumor cells [10].

The involvement of the PPAR pathway and its downstream target genes in COAD remains to be discovered. In this study, we found that the PPAR pathway and downstream target genes were generally repressed in colon cancer tissues through microarray and RNA-seq data. Furthermore, we found a significant correlation between the expression of PPAR pathway-related genes in cancer tissues and the prognosis of COAD patients. We can predict the prognosis of patients as well as their sensitivity to certain chemotherapy medicines based on the expression of these genes. This finding will bring great possibilities for COAD patients' prognosis prediction and accurate postoperative therapy.

## 2. Materials and Methods

### 2.1. Colon Adenocarcinoma Dataset Acquisition

Four datasets (GSE6988, GSE14297, GSE15781, and GSE44076) containing gene expression data and associated clinical data for colon cancer tissues and normal intestinal epithelium were downloaded from the Gene Expression Omnibus (GEO; http://www.ncbi.nlm.nih.gov/geo/). Normalized data were used for analysis the PPAR pathway and downstream gene expression patterns between the normal and tumor tissue. A total of 456 COAD RNA-seq data were downloaded from the TCGA data portal (https://portal.gdc.cancer.gov/) [11]. Six datasets comprising prognostic information of COAD patients were used as the external validation cohorts (GSE12945, GSE17536, GSE33133, GSE39084, GSE39582, and GSE103479) were also downloaded from the GEO.

### 2.2. Pathway Enrichment Analysis

Differentially expressed genes between colon cancer tissues and normal intestinal epithelium were determined by using the R package “limma.” Then, the “clusterProfiler” package [12] in R was used to perform the gene set enrichment analysis (GSEA). We selected the four pathways most closely associated with PPARs for analysis (KEGG_PPAR_SIGNALING_PATHWAY, PPAR-alpha target genes, PPAR-delta target genes, and PPAR-gamma target genes). The pathway gene sets were retrieved from the Molecular Signature Database (MSigDB) [13] or PPAR Gene Database.

### 2.3. PPAR Pathway-Related Gene Procurement

We collected 69 PPAR signaling pathway genes in the Kyoto Encyclopedia of Genes and Genomes (KEGG) database under the name KEGG_PPAR_SIGNALING_PATHWAY. Furthermore, 144 PPAR target genes (40 PPAR-alpha target genes, 64 PPAR-delta target genes, and 69 PPAR-gamma target genes) were downloaded from the PPAR Gene Database (http://www.ppargene.org/). Together, we got 180 PPAR signaling pathway-related genes with prognostic information by intersection. Finally, we removed the low expression genes (FPKM < 1) and obtained 140 PPAR signaling pathway-related genes (PPAR-related genes) for further analysis.

### 2.4. Prognosis Analysis

To find the genes associated with COAD prognosis among the 140 genes involved in the PPAR signaling pathway, we first utilized the univariate Cox proportional hazard regression analysis to analyze the overall survival rate (OS) of COAD patients based on the expression levels of these 140 genes. Eventually, multivariate Cox proportional hazard regression analysis was used to determine an optimized prognostic model. We named this scoring model as “PPAR-Riskscore.”

The PPAR-Riskscore of patients with COAD was established by the following equation: PPAR − Riskscore = ∑_*i*_^*n*^*X*_*i*_∗*Y*_*i*_, where *X*_*i*_ indicates the expression value of gene *i*; meanwhile, *Y*_*i*_ means the coefficient of gene *i* generated from the multivariate Cox regression analysis. According to the median value of the risk score, the samples were classified into two groups (high- and low-risk groups). We employed a time-dependent receiver operating characteristic (ROC) curve to assess the multigene marker's specificity and sensitivity in predicting the 1-year, 3-year, and 5-year OS of COAD and compared them to other clinical indications, like age, sex, subdivision, and TNM stage (II and III). This multigene marker's predictive ability was ultimately confirmed in the GSE12945, GSE17536, GSE33133, GSE39084, GSE39582, and GSE103479 cohorts.

### 2.5. GSVA and Tumor-Infiltrating Immune Cell Analysis

To investigate the relationship between the PPAR-Riskscore and biological pathways, single-sample gene set enrichment analysis (ssGSEA) was used using the “GSVA” R package to analyze the related gene expression patterns of these samples. A score corresponding to each function, as well as the correlation between these functions and the risk score, was calculated for each sample. For GSVA, the gene set file “http://c2.cp.kegg.v7.3.symbols. gm” was obtained from MSigDB. The significance threshold was set at FDR < 0.05. Furthermore, taking into account the significance of the tumor immune microenvironment, the CIBERSORT algorithm was utilized to evaluate the infiltration of 22 types of immune cells.

### 2.6. Chemotherapy Sensitivity Prediction

We utilized the GDSC database to determine the half-maximal inhibitory concentration (IC50) of each COAD patient chemotherapy medications for predicting chemotherapy drug sensitivity using the package to examine the difference in chemotherapy sensitivity between various groups. Statistical significance was defined as a *P* value < 0.05.

### 2.7. Construction of a Nomogram

To find out whether the PPAR-Riskscore was an independent prognostic factor in patients with COAD, we used univariate and multivariate Cox regression analysis methods. A nomogram consisting of a risk score and TNM stage was created based on the findings of multivariate analysis for predicting1-, 3-, and 5-year OS. And the calibration plots were used to evaluate the accuracy between the true OS and the nomogram-predicted values.

### 2.8. Statistical Analyses

To compare normally distributed data, the Student's *t*-test or one-way ANOVA test was utilized. The Mann-Whitney *U* test or the Kruskal-Wallis test was used to access nonnormally distributed data. The nomogram-predicted was built using the “pRRophetic” package “rms” and Iasonos' guide. R software (version 4.0.3) or GraphPad Prism 6.0 was used to conduct all statistical tests and visual analysis.

## 3. Results

### 3.1. Gene Set Enrichment Analysis of PPAR Pathways between Colon Cancer Tissue and Normal Intestinal Epithelium

**Figure 1:**
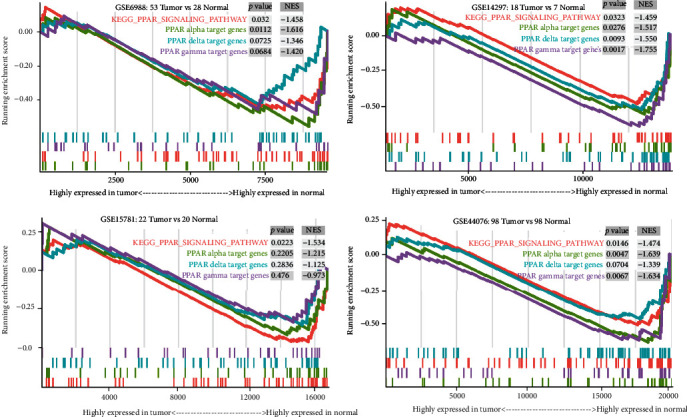
GSEA analyzes the difference in expression levels between colon cancer and normal controls. Four gene sets related to expression levels from four GEO cohorts were analyzed. The curve above the enrichment score of 0 points shows that the gene set is activated in colon cancer. A curve below 0 point shows that it is more active in the normal controls than in colon cancer. *P* adjust: adjusted *P* value; NES: normalized enrichment score.

We selected four PPAR-related pathways from MSigDB to investigate the status of PPAR pathway in colon cancer, including the “KEGG PPAR SIGNALING PATHWAY,” “PPAR-alpha target genes,” “PPAR-delta target genes,” and “PPAR-gamma target genes.” The alterations in these pathways were examined in the four microarrays: GSE6988: 53 COAD vs. 28 normal, GSE14297: 18 COAD vs. 7 normal, GSE15781: 22 COAD vs. 20 normal, and GSE44076: 98 COAD vs. 98 normal. The results of GSEA showed that the four pathways were significantly downregulated in the colon cancer tissue but normal epithelium (Figure 1). Among them, the PPAR-alpha targets was the most significantly downregulated (in which NES value was the smallest among three cohorts, and its *P* value was statistically significant in three of the four cohorts). On the other hand, PPAR-delta targets were not statistically significant in three of the four cohorts, and the inhibitory trend of PPAR-gamma target was not obvious. According to the findings, the PPAR pathway and its target genes are frequently downregulated in colon cancer. We may be able to uncover better treatment targets and prognosis indicators by studying these genes.

### 3.2. The Transcription of PPAR-Related Genes Is Related to the Prognosis of UCEC Patients

**Figure 2:**
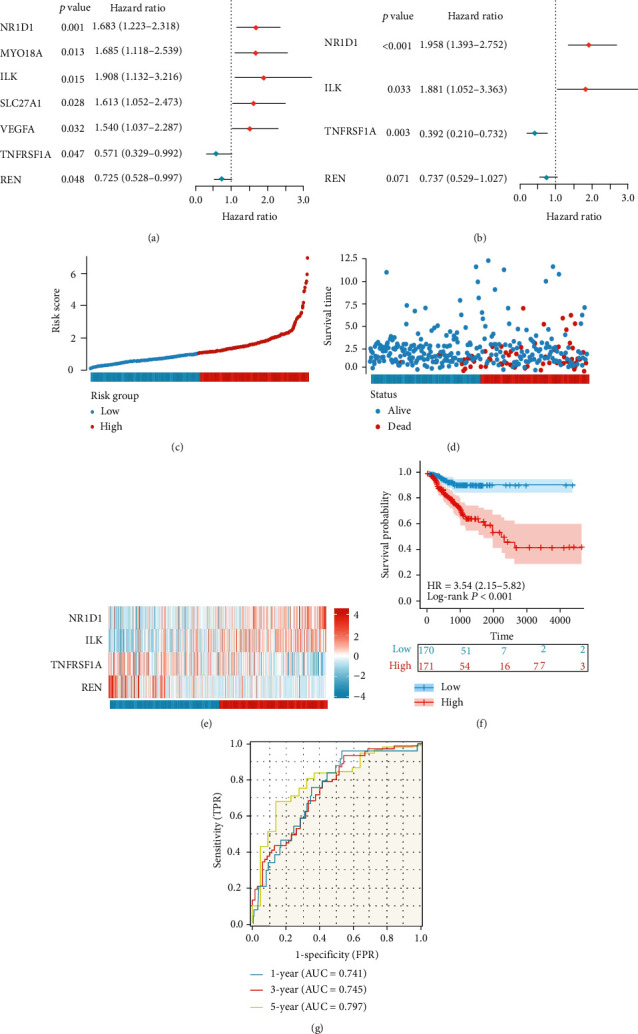
Construction of the PPAR-Riskscore prediction panel. (a) Identification of the prognostic PPAR-related genes by univariate Cox regression analysis; (b) identification of 4 prognostic PPAR-related genes by multivariate Cox regression analysis. (c) and (d) The distribution of PPAR-Riskscore and the survival status of patients with different scores. (e) Heatmap of the expression profiles of the genes in the prognostic signature. (f) The Kaplan-Meier curves of overall survival for patients between the high-risk group and low-risk group. (g) Time-independent receiver operating characteristic (ROC) analysis for evaluating the predictive performance of PPAR-Riskscore.

Cox analysis was performed on the 140 PPAR-related genes in order to identify PPAR-related genes that were associated with OS in the TCGA dataset. Firstly, using univariate Cox regression analysis, 7 PPAR-related genes showed a strong connection with the outcomes of patients with COAD (Figure 2(a)). Then, to ensure that 7 prognostic genes were reliable, multivariate cox univariate regression analysis was used to filter genes without overfitting. Eventually, we created a scoring system named PPAR-Riskscore to predict the prognosis time of COAD patients based on the correlation coefficient of each gene (Figure 2(b)).

(1) $$\begin{array}{c} \text{PPAR}-\text{riskscore}=0.67\times \text{ExpNR}1\text{D}1+0.63\times \text{ExpILK}-0.94\times \text{ExpTNFRSF}1\text{A}-0.31\times \text{ExpREN}. \end{array}$$

Patients were then split into high-risk and low-risk groups based on the median PPAR-Riskscore. The distribution of PPAR-Riskscore and patient survival status is shown in Figures 2(c) and 2(d). NR1D1 and ILK were found to be highly expressed in the high-risk group, whereas TNFRSF1A and REN were found to be strongly expressed in the low-risk group, according to a heatmap of the expression patterns of four genes (Figure 2(e)). The Kaplan-Meier (K-M) curve revealed that the survival rate of patients in the low-risk group was considerably greater than that of patients in the high-risk group (Figure 2(f)). The area under the curve (AUC) values for 1-year, 3-year, and 5-year survival of PPAR-Riskscore in the TCGA dataset were 0.741, 0.745, and 0.797, respectively, according to the time-dependent ROC analysis, consistent with satisfactory model performance (AUC > 0.5; Figure 2(g)). As such, these results indicated that we can successfully establish a PPAR-related COAD prognosis model with certain applicability, among which NR1D1 (nuclear receptor subfamily 1 group D member 1), ILK (integrin-linked kinase), TNFRSF1A (the tumor necrosis factor receptor superfamily, member 1A), and REN (renin) can be effective prognostic factors of COAD patients.

### 3.3. Independent Prognostic Value of the PPAR-Riskscore

**Figure 3:**
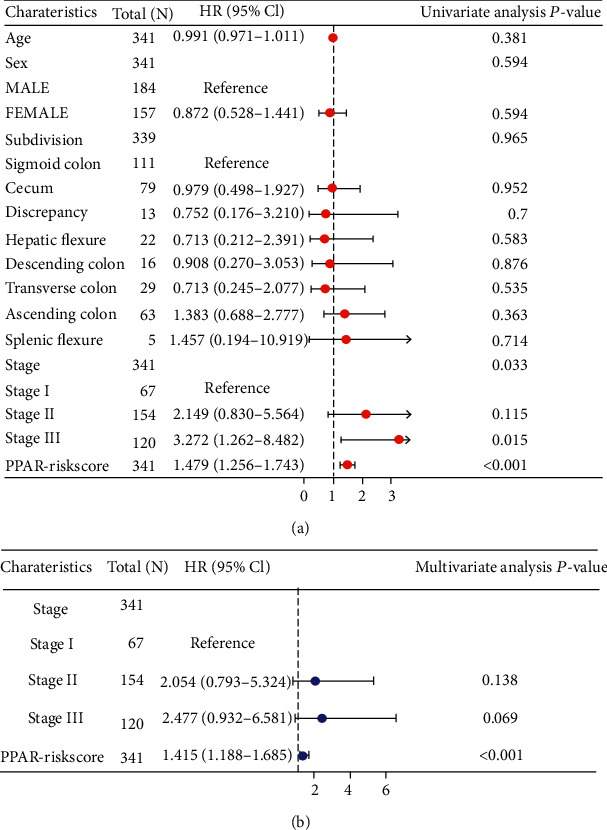
Verification of the independent prognostic value of the PPAR-Riskscore. (a) Forest plots of the univariate Cox regression analyses among PPAR-Riskscore and clinical factors. (b) Forest plots of the multivariate Cox regression analyses among PPAR-Riskscore and clinical factors.

After that, we looked at the association between COAD patient clinical characteristics, risk score, and outcomes in the TCGA cohort to corroborate the PPAR-Riskscore-independent prognostic value. Using univariate Cox regression analysis, it was shown that TNM stage (II and III) and risk score were both substantially associated with patient survival (*P* < 0.001) (Figure 3(a)). Other clinical indicators and OS had some correlations, but they did not achieve statistical significance. These 3 variables were later added as covariates in a multivariate Cox regression analysis, which revealed that the PPAR-Riskscore was a significant independent risk factor for the OS of COAD patients (Figure 3(b)). The predictive power of PPAR-Riskscore (HR = 1.415, 95% CI = 1.188-1.685, *P* < 0.001) was even higher than TNM stage II and stage III. PPAR-Riskscore (HR = 1.415, 95% CI = 1.188-1.685, *P* < 0.001) was an independent predictive factor for COAD patient OS.

### 3.4. External Verification of the PPAR-Riskscore

**Figure 4:**
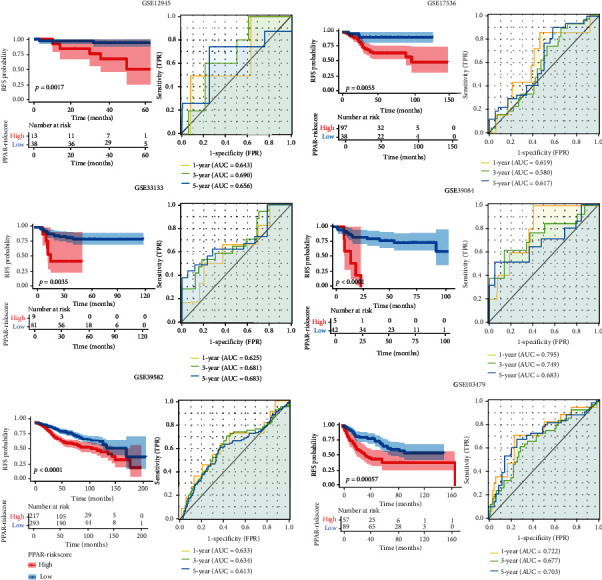
Results of PPAR-Riskscore external validation utilizing six microarray cohorts. Based on the value of PPAR-Riskscore, each cohort was separated equally into high-risk and low-risk groups. Each cohort's Kaplan-Meier analysis and time-dependent receiver operating characteristic curves are shown.

For the purpose of determining if the PPAR-Riskscore is applicable to other COAD cohorts, we utilized external data to validate our findings. To confirm the accuracy of the analytical findings, we enrolled six cohorts, each with more than 50 samples (GSE12945, GSE17536, GSE33133, GSE39084, GSE39582, and GSE103479). Based on PPAR-Riskscore, we used the same procedure to split patients into high-and low-risk groups. Unsurprisingly, patients in the high-risk group had a higher mortality rate in all validation cohorts (*P* < 0.001) (Figure 4). In addition, we use PPAR-Riskscore to predict patients' 1-, 3-, and 5-year OS. PPAR-Riskscore's AUC was 0.6 or higher in most ROC analyses. According to the findings, PPAR-Riskscore has some specific practical application value in predicting COAD patient prognosis.

### 3.5. Identify the Biological Characteristics of Patients with Different PPAR-Riskscore

**Figure 5:**
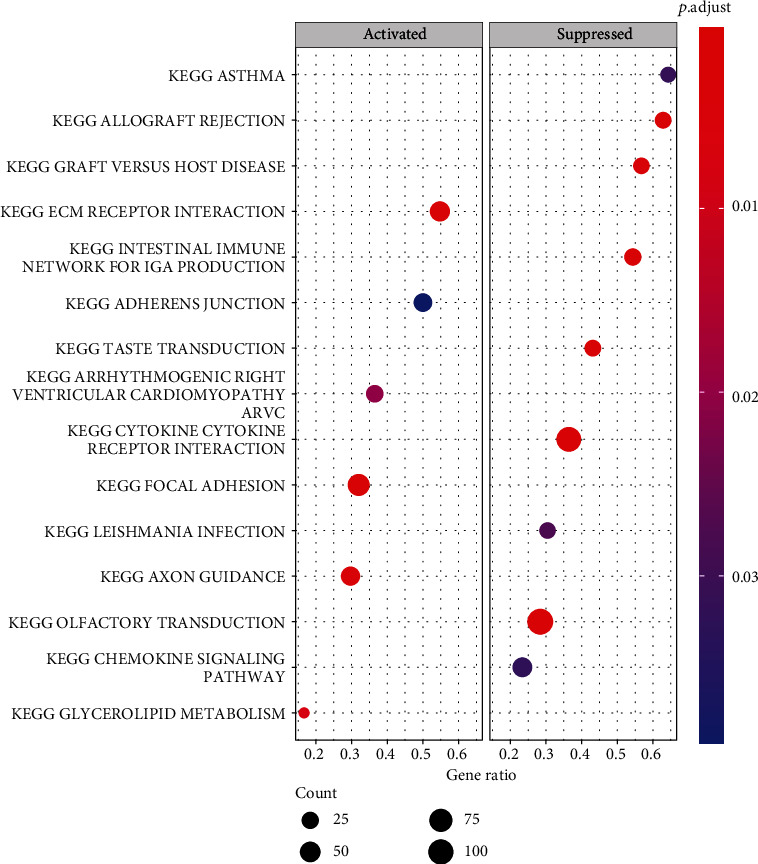
GSEA analysis between patients with high-risk and low-risk PPAR-Riskscore.

To further assess the biological behavior characteristics of patients with varying PPAR-Riskscore, we used GSVA enrichment analysis. In patients with a high PPAR-Riskscore, glycolipid metabolism, ECM-receptor interaction, axon guidance, and focal adhesion were significantly activated compared to patients with a low PPAR-Riskscore (Figure 5). It appears that patients with a high PPAR-Riskscore were more likely to be associated with cancer-related signaling pathways, whereas olfactory transduction and cytokine receptor interaction were highly enriched in patients with a low PPAR-Riskscore and were more likely to be associated with other signaling pathways.

### 3.6. Differences in Infiltrating Immune Cells in Tumor Tissues of Patients with Varying PPAR-Riskscore

**Figure 6:**
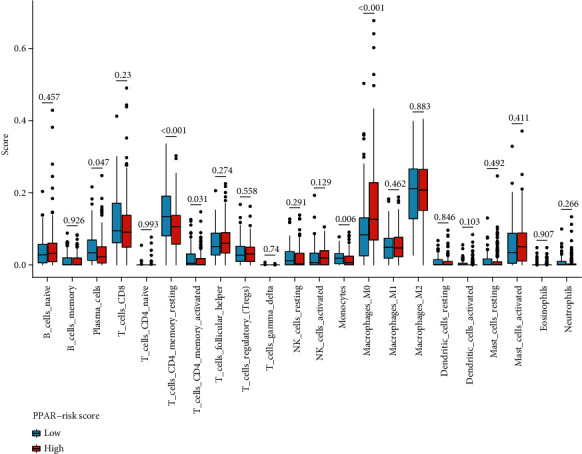
Boxplot compared the expressions of 22 immune cells infiltrating between the high- and low-risk Riskscore via the CIBERSORT algorithm. Each red blot signified the high-risk scores, and each blue dot represents the low-risk groups.

When it comes to immune cell infiltration, the CIBERSORT algorithms were employed to investigate if the PPAR-Riskscore could accurately describe the features of the tumor microenvironment (Figure 6). The results revealed that patients with high PPAR-Riskscore had significantly higher proportions of M0 macrophages, while patients with low PPAR-Riskscore had obviously higher proportions of T cell CD4 memory resting. It is well known that tumor-associated macrophages (TAMs) have protumorigenic properties. In addition, many studies have been proved that activated memory CD4 T cells were a key tool for tumor healing. It can kill cancer cells directly or indirectly by stimulating and recruiting CD8 T cells and various other immune cells [14]. It shows that the PPAR-Riskscore, via controlling immune cell infiltration, may play a role in the occurrence and development of COAD.

### 3.7. Chemotherapeutic Response Analysis

**Figure 7:**
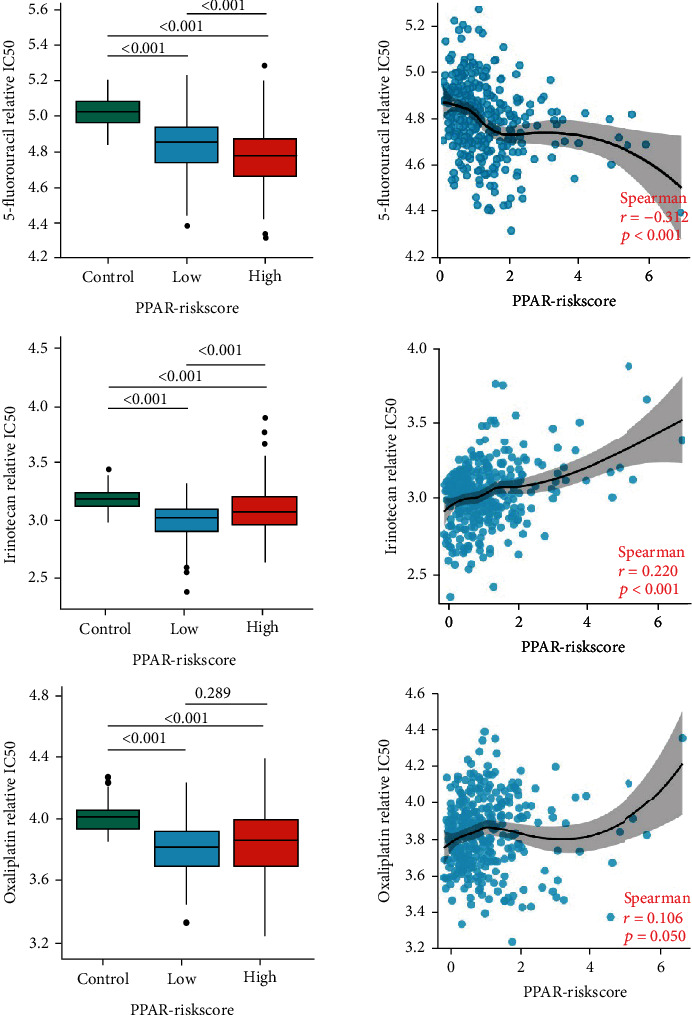
Differences in sensitivity of patients with different PPAR-Riskscore to chemotherapy. The box plots of the estimated IC50for three commonly used chemotherapy drugs and correlation analysis between IC50 of these drugs and PPAR-Riskscore.

Adjuvant chemotherapy is the primary postoperative treatment approach for COAD patients; thus, we studied whether the PPAR-Riskscore could be used to predict the sensitivity of high- and low-risk patients to three chemotherapy medications that are often used for COAD patients (Figure 7). Based on the PPAR-Riskscore, all COAD samples were divided into two groups: those at high risk and those at low risk. Following an analysis of the GDSC database, it was discovered that the IC50 values of commonly used chemotherapy drugs including 5-fluorouracil, irinotecan, and oxaliplatin were elevated in the control group compared to those with COAD groups. The association between the IC50 of these three medications and the PPAR-Riskscore was then investigated using correlation analysis. The results showed that the higher the PPAR-Riskscore, the lower the IC50 of 5-fluorouracil (*R* = −0.312). It showed that 5-fluorouracil could be an effective drug for high PPAR-Riskscore patients.

### 3.8. Construction of a Prognostic Nomogram

**Figure 8:**
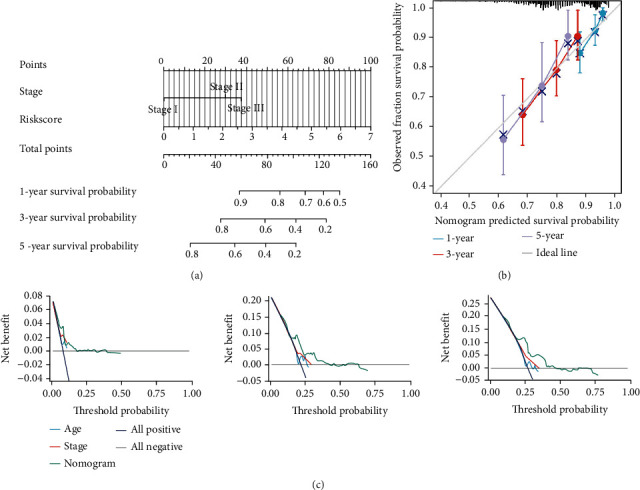
The establishment and verification of a PPAR-Riskscore-based prognostic nomogram. (a) A nomogram for predicting 1-, 3-, and 5-year survival possibilities of patients with COAD. (b) Plots depict the calibration of the nomogram based on risk score in terms of consistency between predicted and observed 1-, 3-, and 5-year outcomes. (c) Decision curve analyses of the nomogram for 1-, 3-, and 5-year risk.

After that, we developed a nomogram incorporating TNM stage and PPAR-Riskscore as two independent prognostic indicators related to COAD patient 1-, 3-, and 5-year OS. This allowed us to evaluate the ability of PPAR-Riskscore to reliably predict the clinical prognosis and facilitate clinical usage of COAD (Figure 8(a)). The prognosis of a patient may be computed by adding up the contribution scores of each item in the equation. As shown in Figure 8(b), calibration plots for the surviving time points were produced, demonstrating that the nomogram had excellent prediction ability. The results of the decision curve study show that the clinical applicability of our nomogram surpassed the clinical features by a significant margin (Figure 8(c)). It showed that by combining the PPAR-Riskscore with clinical parameters to predict prognosis, a greater number of patients might benefit from it.

## 4. Discussion

PPARs have gone from being totally unknown receptors to key actors in a variety of physiological processes and pathological situations in the past twenty years. The role of these receptors in cell differentiation and cancer is one of their most important functions. Numerous reports have indicated that PPARs act as tumor suppressors or tumor accelerators, indicating that they might be used as pharmacological targets for cancer prevention and therapy [15–21]. The related influence of PPAR on tumor development has always been related to cell cycle inhibitory genes such as p18, p21, and p27. It can induce apoptosis by inhibiting B-cell lymphoma-2 (Bcl-2), and it can also reduce angiogenesis by inhibiting vascular endothelial growth factor (VEGF) [22, 23]. PPAR*α* is expressed in hepatocytes, cardiomyocytes, proximal tubular cells, and brown adipose tissue [24]. PPAR*β*/*δ* is expressed in several tissues with some species differences, while PPAR*γ* is expressed in white and brown adipose tissue, the gut, and immune cells and is related to adipogenesis, lipid storage, and glucose homeostasis [25]. Recently, a study showed that PPAR expressions are explicitly deregulated in colorectal cancer (CRC), with PPAR*α* and PPAR*δ* being overexpressed, while PPAR*γ* is suppressed in CRC tumor tissues. More importantly, abnormal PPAR expression levels in tumor tissues seemed to be linked to CRC development and poor prognosis [26]. Therefore, we need to find biomarkers that that reliably predict the prognosis of COAD patients in order to guarantee that patients get more suitable and successful therapy.

To find the COAD prognostic indicators linked with PPAR, 140 genes implicated in the PPAR signaling pathway were investigated in this research utilizing COAD patient data obtained by bioinformatics approaches. Subsequently, we determined the novel four gene models (NR1D1, ILK, TNFRSF1A, and REN) and established a prognostic model named PPAR-Riskscore. Using the risk score, COAD patients are divided into two groups: those at high risk and those at low risk. A survival curve analysis reveals that patients in the high-risk group have considerably poorer outcomes than those in the low-risk group. The ROC curve demonstrates that this model has excellent 1-, 3-, and 5-year survival prediction accuracy. According to multivariate Cox regression analysis, PPAR-Riskscore is an independent risk factor for COAD.

Among the genes of PPAR-Riskscore, NR1D1 is the most studied, and it is the target of PPAR-gamma-related pathway. Nuclear receptor subfamily 1 group D member 1 (NR1D1; REV-ERB*α*) is a nuclear receptor that controls a variety of physiological functions [27]. It is also thought to have a role in cancer. Many malignancies are killed by pharmacological activation of NR1D1 [28]. One research found that NR1D1 interferes with the recruitment of DDR complexes to damaged DNA locations, hence impairing normal DNA repair. Aside from this, it was shown that NR1D1 improved the susceptibility of breast cancer cells to DNA damage-induced chemotherapy, hence increasing the likelihood of PCR in breast cancer patients [29]. Another research has shown that NR1D1 inhibits activation of the JAK/STAT3 signaling pathway by upregulating the expression of SOCS3, thus suppressing ovarian cancer cell growth and inducing apoptosis. Through the use of immunohistochemistry and other techniques, X. Wang et al. discovered that the expression of REV-ERB was reduced in gastric cancer tissue. In gastric cancer, the researchers discovered that REV-ERB expression was substantially related with poor differentiation, TMN stage, and lymph node metastasis. Furthermore, it has been shown that REV-ERB expression is substantially associated with patient survival time, suggesting that REV-ERB may be a prognostic factor in gastric cancer on an independent basis [30].

ILK, a target of PPAR*β*/*δ*-related pathway, is a 59 kDa serine/threonine protein kinase that binds to the cytoplasmic domains of integrins *β*1 and *β*3 and is distributed in the cytoplasm near the cell membrane. In the process of carcinogenesis, progression, and metastatic processes, it has dual effects on the cells. A number of signal transduction pathways are regulated by ILK, which also forms a scaffold complex with cytoskeleton proteins, all of which are critical in the regulation of cell motility, tumor growth, and invasion [31]. ILK was recently discovered to be overexpressed in several cancers (the prostate [32], ovary [33], breast [34], colon [35], lung [36], and thyroid [37]), contributing to their proliferation, invasion, and metastasis by regulating EMT-related genes. EMT is a key process that drives cancer occurrence and progression. Currently, it has been documented that ILK is associated with colorectal cancer. It has been shown in pathological results that high ILK expression levels are associated with CRC stage, lymph node metastasis, and patient survival [38]. The in vitro CRC studies by Shen et al. also revealed that overexpression of ILK may stimulate the development of EMT in CRC cells [35]. In addition, ILK expression is upregulated in ovarian cancer, and it has a positive correlation with tumor development. The in vivo tumorigenesis of human ovarian cancer cells is suppressed by silencing the ILK gene [33]. On the contrary, it has been reported that loss of ILK abrogates the mechanosensing capability of breast cancer cells and blocks tumorigenic and metastatic potential [34].

TNF-*α* and REN are also a target of PPAR-gamma-related pathway, and TNF-*α* is a highly active cytokine engaging in the signaling pathway of necrosis or apoptosis in cells [39]. TNF-*α* is an endogenous tumor promoter that is generated by neoplastic cells or cells in the tumor microenvironment. TNFRSF1A is considered the dominant signaling receptor for the cytokine TNF-*α*. When TNF-*α* binds to TNFRSF1A, it activates the transcription factor NF-B, mediates apoptosis, and regulates inflammation [40]. In the study by Yang et al., identification of TNFRSF1A by bioinformatics analyses such as WGCNA showed that the expression of TNFRSF1A was highly expressed in glioma samples compared with normal brain samples. In addition, the expression level of TNFRSF1A correlated with WHO grade and other clinical parameters, and it was revealed to be an independent predictive factor. Knockdown of TNFRSF1A inhibited the proliferation and migration of glioma cell lines in vitro. These findings suggest that TNFRSF1A may be a promising biomarker for the diagnosis, treatment, and prognosis of mesenchymal subtype gliomas [41]. As a result of the disruption of REN tumor suppression function shown in human medulloblastoma, the issue of whether this gene is involved in the formation of cerebellar GCPs has been raised. Through overexpression and function knockout studies, Argenti et al. demonstrated that REN promotes growth stagnation, differentiation, and apoptosis and showed that loss of REN may release inhibition of the Hedgehog pathway and promote tumorigenesis.

According to the information presented above, the target gene used to construct the model in this research has gotten varying degrees of attention and investigation for various kinds of cancers. PPAR-Riskscore is an independent prognostic factor (Figure 3). The tool is shown to be able to further predict survival probability in patients with the same tumor stage, so it has a high level of clinical value. We conducted gene set variation analysis (Figure 5) to understand the biological characteristics of patients with different PPAR-Riskscore. The results showed that pathways such as glycolipid metabolism, ECM-receptor interaction, axon guidance, and focal adhesion were significantly upregulated in patients with high PPAR-Riskscore. Additionally, we developed a nomogram to improve prognosis accuracy and make clinical application easier. For cancer prognosis, nomograms are widely used. Using statistical methods, it combines parameters to predict patients' prognosis. Calibration charts and decision curves were used to analyze our nomogram. The results showed that the nomogram was more accurate and could benefit more patients than simply using one factor.

It is worth noting that the research presents limitations: (1) the transcriptome data utilized in the model was obtained through sequencing. Parameters may need to be adjusted if microarrays and qPCR are used to calculate PPAR-Riskscore. (2) An appropriate cut-off value needs to be determined with a larger cohort. (3) The patient population is heterogeneous in this retrospective analysis. Therefore, more clinical investigations are needed to verify the efficiency of the prediction tools and nomogram developed in this study.

## 5. Conclusions

In conclusion, our PPAR gene expression-based scoring system is a valuable tool for predicting COAD patient survival. It can also aid therapeutic chemotherapy by evaluating the score. However, more clinical trials are needed to corroborate our findings.

## Data Availability

The data used to support the findings of this study are available from the corresponding author upon reasonable request.
